# Deubiquitination of RIPK2 by OTUB2 augments NOD2 signalling and protective effects in intestinal inflammation

**DOI:** 10.1002/ctm2.70038

**Published:** 2024-10-02

**Authors:** Xue Du, Jun Xu, Fuqi Mei, Jiangyun Shen, Bincheng Zhou, Zhenhu Zhu, Zhongding Li, Xian Su, Jianmin Li, Dirk Schlüter, Jing Ruan, Xu Wang

**Affiliations:** ^1^ School of Pharmaceutical Sciences Wenzhou Medical University Wenzhou China; ^2^ Oujiang Laboratory (Zhejiang Lab for Regenerative Medicine, Vision and Brain Health) Wenzhou China; ^3^ Department of Pathology The First Affiliated Hospital of Wenzhou Medical University Wenzhou China; ^4^ Hannover Medical School Institute of Medical Microbiology and Hospital Epidemiology Hannover Germany

**Keywords:** inflammatory bowel disease, OTUB2, RIPK2, signal transduction, ubiquitination

## Abstract

**Background:**

Inflammatory bowel disease (IBD) is a chronic inflammatory disorder of the gastrointestinal tract, but the molecular mechanisms underlying IBD are incompletely understood. In this study, we explored the role and regulating mechanism of otubain 2 (OTUB2), a deubiquitinating enzyme, in IBD.

**Methods:**

To study the function of OTUB2 in IBD, we generated *Otub2* ^–/–^ mice and treated them with dextran sulfate sodium (DSS) to induce experimental colitis. Bone marrow transplantation was performed to identify the cell populations that were affected by OTUB2 in colitis. The molecular mechanism of OTUB2 in signal transduction was studied by various biochemical methods.

**Results:**

OTUB2 was highly expressed in colon‐infiltrating macrophages in both humans with IBD and mice with DSS‐induced experimental colitis. Colitis was significantly aggravated in *Otub2* ^–/–^ mice and bone marrow chimeric mice receiving *Otub2* ^–/–^ bone marrow. OTUB2‐deficiency impaired the production of cytokines and chemokines in macrophages in response to the NOD2 agonist muramyl dipeptide (MDP). Upon MDP stimulation, OTUB2 promoted NOD2 signaling by stabilizing RIPK2. Mechanistically, OTUB2 inhibited the proteasomal degradation of RIPK2 by removing K48‐linked polyubiquitination on RIPK2, which was mediated by the active C51 residue in OTUB2. In mice, OTUB2 ablation abolished the protective effects of MDP administration in colitis.

**Conclusion:**

This study identified OTUB2 as a novel regulator of intestinal inflammation.

## Graphical abstract

- Global knockout or haematopoietic cell‐specific deficiency of OTUB2 aggravates experimental colitis in mice.
- OTUB2 is highly expressed in macrophages in the inflamed intestinal mucosa of both humans and rodents.
- OTUB2 potentiates MDP‐induced physiological inflammation by K48 deubiquitinating RIPK2.

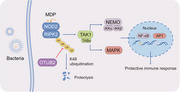

## 1 INTRODUCTION

Inflammatory bowel disease (IBD) is a long‐standing inflammatory disorder of the digestive tract and is predominantly represented by Crohn's disease (CD) and ulcerative colitis (UC).^1^, ^2^ IBD is pathologically characterised by epithelial destruction, stromal cell proliferation, and immune activation in the intestine and causes multiple symptoms such as weight loss, fatigue, diarrhoea, abdominal pain, and rectal bleeding.^3^ Accumulating evidence suggests that IBD is a complex multifactorial disease with various environmental, microbial, immunological, and genetic factors contributing to its etiology and pathogenesis.^4^, ^5^ Given that the intestine provides a key interface for immune cells to detect and respond to the dense and complex enteric microbiota, an aberrant immune response to intestinal microbes by mucosal immune cells may instigate IBD in a genetically susceptible person.^6^

Immune responses are tightly regulated by ubiquitination, a post‐translational modification catalysed by an enzymatic cascade comprising ubiquitin‐activating enzymes (E1s), ubiquitin‐conjugating enzymes (E2s), and ubiquitin ligases (E3s).^7^ Of note, ubiquitination is a reversible process and it can be counter‐regulated by deubiquitinating enzymes (DUBs), which have emerged as critical regulators of intestinal immunity.^8^ A20, which is encoded by the IBD susceptibility gene *Tnfaip3*, is a DUB that regulates both innate and adaptive immune responses.^9^, ^10^, ^11^, ^12^ Mice lacking A20 specifically in dendritic cells, macrophages, or intestinal epithelial cells are predisposed to colitis.^13^, ^14^, ^15^, ^16^ Besides, colitis can also be inhibited by USP7 and USP8, two DUBs that restrain intestinal inflammation by enhancing the immunosuppressive function of regulatory T cells.^17^, ^18^ Another DUB, OTUD1, ameliorates dextran sulphate sodium (DSS)‐induced colitis in mice by inhibiting RIPK1‐mediated cytokine production in innate immune cells.^19^ Moreover, the DUB OTUD5 has been shown to ameliorate experimental colitis by reducing type I interferon production.^20^ Hence, DUBs are critical for the immunomodulatory function of immune cells during intestinal inflammation.

Like A20 and OTUD1, otubain 2 (OTUB2) is a DUB of the ovarian tumour protease subfamily.^21^ Recent studies have found that OTUB2 regulates tumourigenesis and/or progression of multiple cancers.^22^, ^23^, ^24^ For example, OTUB2 deubiquitinates and stabilises U2AF2 and thereby promotes tumourigenesis of non‐small cell lung cancer.^23^ Besides, OTUB2 promotes breast cancer metastasis by deubiquitinating and activating YAP/TAZ.^24^ OTUB2 has also been shown to inhibit antitumour immunity by disrupting the ubiquitination and degradation of PD‐L1 on tumour cells.^25^ In sharp contrast, OTUB2 inhibits tumourigenesis and progression of tongue and oesophageal squamous cell carcinoma by mediating the deubiquitination and activation of STAT1.^22^ Upon virus infection, OTUB2 inhibits type I IFN production by deubiquitinating TRAF3 and TRAF6, resulting in impaired cellular antiviral response.^26^ Moreover, OTUB2 has also been shown to fine‐tune DNA repair by suppressing RNF8‐mediated ubiquitination in response to DNA double‐strand breaks.^27^ However, the pathophysiological function of OTUB2 in intestinal inflammation remains unknown.

In this study, we found that ablation of OTUB2 aggravated DSS‐induced experimental colitis and OTUB2 alleviated colonic inflammation mainly by regulating haematopoietic cells. In both humans and mice, OTUB2 was highly expressed in colon‐infiltrating macrophages and it was required for NOD2‐mediated protective effects in macrophages. Mechanistically, OTUB2 enhanced NOD2 signalling by stabilising RIPK2 through K48 deubiquitination. Altogether, these results identify OTUB2 as an important gatekeeper of intestinal inflammation.

## 2 MATERIALS AND METHODS

### 2.1 Mice

*Otub2* ^–/–^ mice on the C57BL/6 background were generated by Cyagen Biosciences (Suzhou, China). Briefly, exons 3 and 4 of *Otub2* were ablated in *Otub2* ^–/–^ mice (Figure S1A). Heterozygous *Otub2* ^±^ mice were bred to generate *Otub2* ^–/–^ and *Otub2* ^+/+^ littermates. Genotyping was carried out by PCR on tail DNA (primer 1: F1, 5′‐CACCCCAGGCCTAGTAAAGAAG‐3′, R1, 5′‐TAACACCAGCCTGCTCACCTATC‐3′; primer 2: F2, 5′‐TAGGCAACATTGGGGTGGGCAC‐3′, R1, 5′‐TAACACCAGCCTGCTCACCTATC‐3′) (Figure S1A and B). The mice used for experiments were kept in a specific‐pathogen‐free environment in the Laboratory Animal Resources Center of Wenzhou Medical University. Animal studies were conducted in accordance with animal management regulations and approved by the Animal Management and Ethics Committee of Wenzhou Medical University (approval number: wydw2023‐0598).

### 2.2 DSS‐ and TNBS‐induced colitis

Gender‐ and age‐matched *Otub2* ^–/–^ and *Otub2* ^+/+^ mice (8 to 12 weeks old) were kept together for 2 weeks and then given drinking water containing 2% DSS (Cat#: 160110, MP Biomedicals) for 8 days. After that, they were fed regular drinking water for 2 days. For the colitis model with muramyl dipeptide (MDP) treatment, mice were intraperitoneally injected with 100 µg of MDP (Cat#: tlrl‐gmdp, InvivoGen) in the first 3 days, and mice in the control group were intraperitoneally injected with volume‐matched PBS (Cat#: P1020, Solarbio). For the TNBS‐induced colitis model, 1% TNBS (Cat#: P2297, Sigma‐Aldrich) was evenly applied to the dehaired skin on the abdomen of each mouse. One week later, 100 µL of 2.5% TNBS was injected into the colon of each mouse from the anus with a catheter. Body weight and stool consistency were recorded daily, and fecal occult blood was detected with the fecal occult blood qualitative detection kit (Cat#: SK1030K‐100T, G‐clone, Beijing, China). The disease activity index (DAI) was shown as the sum of three parameters: score of weight loss (0: 0; 1: 1−5%; 2: 5−10%; 3: 10−20%; 4: > 20%), score of stool consistency (0: normal; 1: soft but still formed; 2: very soft; 3: diarrhoea), and score of fecal occult blood (0: negative; 1: positive haemoccult; 2: visible blood traces in stool; 3: rectal bleeding).

### 2.3 Cell culture and treatment

MODE‐K cells (Cat#: BFN608006456) were obtained from Bluefbio (Shanghai, China). RAW264.7 cells (Cat#: SCSP‐5036), L‐929 cells (Cat#: GNM28) and NIH/3T3 cells (Cat#: SCSP‐515) were purchased from the National Collection of Authenticated Cell Culture (Shanghai, China). MODE‐K, NIH/3T3, and RAW264.7 cells were cultured in DMEM medium (Cat#: C11995500BT, Thermo Fisher Scientific) containing 10% fetal bovine serum (FBS; Cat#: F101‐01, Vazyme Biotech) and 1% penicillin/streptomycin (Cat#: P1400, Solarbio). L929 cells were maintained in MEM‐α medium (Cat#: 109C12571500BT, Thermo Fisher Scientific) containing 10% FBS and 1% penicillin/streptomycin. *Otub2* ^–/–^ MODE‐K cells were generated using the CRISPR/Cas9 technology (gRNA sequence: GAACTGCACGATTCGGTCCG). Cells were treated with LPS (Cat#: L2880, Sigma‐Aldrich), L‐18 MDP (Cat#: tlrl‐Imdp, InvivoGen), cycloheximide (CHX; Cat#: 239763‐M, Merck), MG‐132 (CAT#: HY‐13259, MedChemExpress) or chloroquine (CQ; Cat#: HY‐17589A, MedChemExpress) for indicated periods of time before further analysis.

### 2.4 Plasmid transfection

FLAG‐OTUB2, FLAG‐OTUB2 C51S, HIS‐MYC‐RIPK2, HA‐K48 Ub and FLAG‐Vector plasmids were constructed by Genechem (Shanghai, China). Indicated plasmids were transfected into NIH/3T3 cells with Lipofectamine 3000 reagent (Cat#: L3000015, Thermo Fisher Scientific) following the producer's protocols.

### 2.5 Bone marrow‐derived macrophages (BMDMs)

Bone marrow cells were flushed out of the femur and tibia with RPMI 1640 culture medium (Cat#: 11875093, Thermo Fisher Scientific) containing 1% penicillin and streptomycin, and then filtered through a 70 µm filter (Cat#: WHB‐70 µm, WHB, Shanghai, China). Erythrocytes were eliminated by incubation with red blood cell lysis solution (Cat#: R1010, Solarbio). After washing with PBS, cells were cultured in DMEM medium containing 20% L‐929 culture supernatant, 10% FBS, and 1% penicillin/streptomycin for 7 days to generate BMDMs.

### 2.6 Quantitative Real‐Time PCR (qRT‐PCR)

Total RNA from colon tissue or BMDMs was isolated with RNAiso Plus (Cat#: 9109, Takara Bio). Then, cDNA was generated with the PrimeScript™ RT reagent Kit (Cat#: RR047A, Takara Bio). PCR was carried out on a QuantStudio™ 5 Real‐Time PCR System (Thermo Fisher Scientific) using TB Green^®^ Premix Ex Taq™ II (Cat#: RR820A, Takara Bio) and corresponding primers (Table S1).

### 2.7 Bone marrow transplantation

The recipient *Otub2* ^+/+^ mice (male, 6 to 8 weeks old) were fed acidified water containing neomycin (Cat#: HY‐B0470/CS‐2584, MedChemExpress) for 1 week before receiving irradiation at a dose of 8.5 Gy. Bone marrow cells from male *Otub2* ^–/–^ and *Otub2* ^+/+^ mice were counted with a haemocytometer. Within 12 h after irradiation, 5 × 10^6^ bone marrow cells were intravenously infused into each recipient mouse through the tail vein. After transplantation, the recipient mice were given acidified water containing antibiotics for one more week and then rested for 7 weeks to allow haematopoietic reconstitution.

### 2.8 Western blot

RIPA lysis buffer (Cat#: AR0105, Boster Bio) supplemented with phosphatase inhibitor cocktail II (Cat#: HY‐K0022, MedChemExpress) and protease inhibitor cocktail (Cat#: HY‐K0010, MedChemExpress) was used to lyse colon tissue and cultured cells. After protein concentration determination with Quick Start™ Bradford 1 × Dye Reagent (Cat#: 5000205, BIO‐RAD), samples were denatured and then separated by SDS‐PAGE. Subsequently, the samples were transferred to PVDF membranes (Cat#: 10600023, Cytiva), followed by incubation with primary antibodies against OTUB2 (Cat#: NBP2‐03223, Novus Biologicals), OTUB1 (Cat#: NBP1‐49934, Novus Biologicals), NOD2 (Cat #: sc‐56168, Santa Cruz Biotechnology), p‐RIPK2 (Cat#: 14397, Cell Signaling Technology), RIPK2 (Cat#: 4142S, Cell Signaling Technology), XIAP (CAT#: 10037‐1‐Ig, Proteintech), p‐p65 (Cat#: 3033S, Cell Signaling Technology), p65 (Cat#: 8242S, Cell Signaling Technology), p‐JNK (Cat#: 4668S, Cell Signaling Technology), JNK (Cat#: 9252S, Cell Signaling Technology) Technology), p‐p38 (Cat#: 4631S, Cell Signaling Technology), p38 (Cat#: 8690S, Cell Signaling Technology), HA (Cat#: 51064‐2‐AP, Proteintech), FLAG (Cat#: 14793S, Cell Signaling Technology), MYC (Cat#: 16286‐1‐AP, Proteintech), K48 polyubiquitin (Cat#: 8081S, Cell Signaling Technology), or GAPDH (Cat#:60004‐1‐Ig, Proteintech) at 4°C overnight. Then, HRP‐conjugated secondary antibodies were added. Images were developed with the Chemiluminescent Kit (Cat#: P2300, NCM Biotech) and captured by the Fusion FX.EDGE system (Vilber, France).

### 2.9 Immunoprecipitation

A small fraction of protein solution was reserved as input control, and the remaining protein solution was incubated with corresponding antibodies (1 µg antibody/mg protein) under gentle rotation at 4°C overnight. Then, 20 µL of Protein A+G Agarose Beads (Cat#: P2012, Beyotime) was added to capture the immunocomplex. The beads were washed with PBS for 5 times and then boiled in 1× loading buffer for analysis with Western blot.

### 2.10 In vitro deubiquitination assay

FLAG‐OTUB2 or HIS‐MYC‐RIPK2 + HA‐K48 Ub plasmids were transfected into NIH/3T3 cells. Twenty‐four hours after transfection, OTUB2 and K48 ubiquitinated RIPK2 were harvested from transfected cells by immunoprecipitation with anti‐FLAG and anti‐MYC antibodies, respectively. Thereafter, K48 ubiquitinated RIPK2 was incubated with/without OTUB2 in the in vitro deubiquitination buffer (5 mM MgCl_2_, 2 mM ATP‐Na_2_, 2 mM DTT, 5% glycerol, 50 mM Tris‐HCl) at 37°C for 2 h. After incubation, the samples were analysed by Western blot.

### 2.11 Clinical samples

Colon biopsy samples from patients with UC were obtained from the First Affiliated Hospital of Wenzhou Medical University. Besides, tumour‐adjacent normal colon tissue was collected from patients undergoing colectomy and used as control tissue. The study was approved by the Ethics Committee in Clinical Research of the First Affiliated Hospital of Wenzhou Medical University (Approval number: KY2023‐R182) and carried out in accordance with rules of the Declaration of Helsinki. Patient information was summarised in Table S2.

### 2.12 Histological analysis

Animal tissue was fixed with 4% paraformaldehyde for 24 h, followed by dehydration and embedding. Then, the tissue was cut into 5 µm sections. After dewaxing and hydration, sections were subjected to Periodic Acid Schiff/Alcian Blue (PAS/AB; Cat#: G1285, Solarbio) and Haematoxylin and Eosin (H & E; Cat#: G1120, Solarbio) staining following the manufacturer's protocols. Histology scores were shown as the sum of tissue damage score (0, normal morphology; 1, loss of goblet cells; 2, massive loss of goblet cells; 3, loss of crypts; 4, massive loss of crypts) and inflammation score (0, no infiltration; 1, infiltration with individual scattered inflammatory cells; 2, mild infiltration of the muscularis mucosa; 3, extensive infiltration of the muscularis mucosa with substantial oedema; 4, infiltration of the submucosa).

### 2.13 Immunohistochemistry

Tissue sections were prepared as described in ‘Histological analysis’. The sections were incubated with 5% BSA blocking buffer (Cat#: SW3015, Solarbio) at room temperature for 30 min, followed by incubation with the antibody against OTUB2 (Cat#: NBP2‐03223, Novus Biologicals) at 4°C overnight. Subsequently, the secondary antibody was added. Signals were detected with 3,3′‐diaminobenzidine tetrahydrochloride (CAT#: ZLI‐9018, ZSGB‐BIO, Beijing, China), and the nuclei were stained with haematoxylin. The Nikon ECLIPSE Ni‐U microscope (Nikon, Tokyo, Japan) was used to capture images.

### 2.14 Immunofluorescence

Macrophages were immersed in 4% paraformaldehyde for 15 min and subsequently permeabilised with .5% Triton X‐100 for 20 min. Cells were incubated with 5% BSA at room temperature for 30 min, followed by incubation with primary antibodies against OTUB2 (Cat#: NBP2‐03223, Novus Biologicals) and RIPK2 (Cat#:4142S, Cell Signaling Technology) at 4°C overnight. Thereafter, fluorescence‐conjugated secondary antibodies were added and incubated at 37°C for 1 h. Colon sections were prepared as described before in ‘Immunohistochemistry’, and incubated with antibodies against F4/80 (Cat#: 30325S, Cell Signaling Technology) and OTUB2 (Cat#: NBP2‐03223, Novus Biologicals). TUNEL staining was performed using the CoraLite® Plus 488 TUNEL Assay Apoptosis Detection Kit (Cat#: PF00006, Proteintech) according to the producer's protocols. Cell nuclei were stained with DAPI (Cat#: C1006, Beyotime). All images were taken on a ZEISS LSM 980 confocal microscope (Carl Zeiss AG).

### 2.15 Quantification and statistical analyses

Data are displayed as mean ± SEM. The GraphPad Prism 9 software (GraphPad) was applied to perform statistical analyses. The two‐tailed Student's *t* test was applied to analyse the statistical difference between two groups, and the Analysis of Variance (ANOVA) test was used to compare more than two groups of data. The differences were considered statistically significant when *p *< .05. Comparisons and sample sizes are included in figures or figure legends. Blinding was not applied in this study, and mice of the same genotype were assigned to different experimental groups in a random way. Experiments were repeated at least twice.

## 3 RESULTS

### 3.1 OTUB2 expression is downregulated in the colon during colitis

**FIGURE 1:**
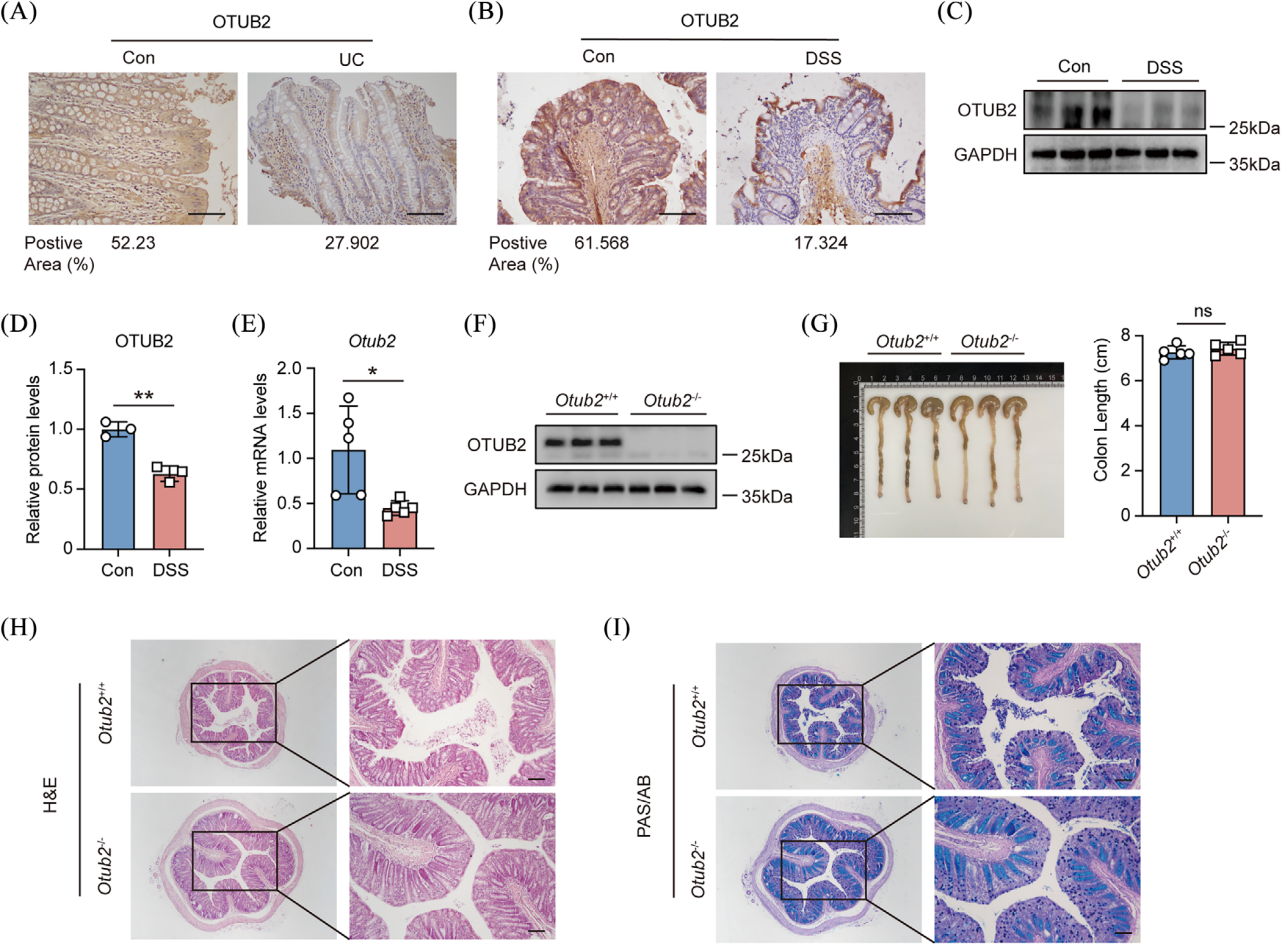
Colonic expression OTUB2 is downregulated in colitis. (A) Representative immunohistochemical staining of OTUB2 in control and UC colon samples. Scale bar = 50 µm. (B) Representative immunohistochemical staining of OTUB2 in colon samples from control and DSS‐treated C57BL/6 mice. Control mice received regular drinking water. Mice in the DSS group were given 2% DSS for 8 days, followed by regular drinking water for 2 days. Scale bar = 50 µm. (C, D) Representative immunoblots (C) and relative quantification (D) of OTUB2 protein in colon samples from control and DSS‐treated C57BL/6 mice. (E) Relative mRNA levels of *Otub2* in colon samples from control and DSS‐treated C57BL/6 mice were analysed by qRT‐PCR. (F) Western blot analysis of OTUB2 protein abundance in colon samples from *Otub2* ^+/+^ and *Otub2* ^–/–^ mice. (G) The representative image (left) and length (right) of colons from *Otub2* ^+/+^ and *Otub2* ^–/–^ mice. (H, I) Representative H&E (H) and PAS/AB (I) staining of colons from *Otub2* ^+/+^ and *Otub2* ^–/–^ mice. Scale bar = 50 µm. Data in D, E and G are shown as mean ± SEM. ns, no significant difference. *p < .05, **p < .01.

To explore the potential role of OTUB2 in intestinal inflammation, we first compared the expression of OTUB2 in normal adjacent colon tissues from colon cancer patients and inflamed colon samples from UC patients. As shown in Figure 1A, OTUB2 expression was strongly reduced in UC colon tissues as compared with normal control colon tissues. In line with this, OTUB2 expression was also significantly reduced in the colon of mice after DSS treatment at both translational and transcriptional levels (Figure 1B‐E), indicating that OTUB2 may ameliorate intestinal inflammation. To study the in vivo function of OTUB2, we generated *Otub2* ^–/–^ mice (Figures 1F and S1). Of note, *Otub2* ^–/–^ mice were born normally and reached adulthood without showing obvious defects. Further analysis revealed that deletion of OTUB2 did not change the body weight of mice and had no impact on the size and architecture of the colon under physiological conditions (Figures 1G‐I and S2).

### 3.2 OTUB2 alleviates DSS‐induced colitis in mice

**FIGURE 2:**
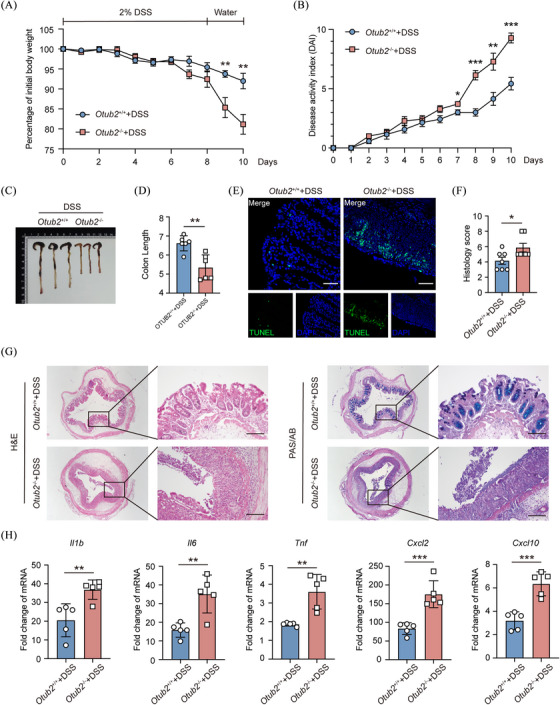
OTUB2 ablation exacerbates DSS‐induced colonic inflammation. (A, B) *Otub2* ^+/+^ and *Otub2* ^–/–^ mice were fed drinking water containing 2% DSS for 8 days, and then given normal drinking water for 2 days. Body weight (A) and disease activity index (B) were recorded daily (*n* = 6/group). (C, D) The representative image (C) and length (D) of colons from *Otub2* ^+/+^ and *Otub2* ^–/–^ mice on day 10 after DSS treatment. (E) Representative TUNEL staining of colons from *Otub2* ^+/+^ and *Otub2* ^–/–^ mice on day 10 after DSS treatment. Scale bar = 50 µm. (F, G) Histology score (F) as well as representative H&E and PAS/AB staining (G) of colons from *Otub2* ^+/+^ and *Otub2* ^–/–^ mice on day 10 after DSS treatment. Scale bar = 50 µm. (H) The colonic transcription levels of *Ilb*, *Il6*, *Tnf*, *Cxcl2* and *Cxcl10* mRNA were analysed by qRT‐PCR. Data are presented as the relative increase over untreated control samples. Data in A, B, D, E and G are shown as mean ± SEM. *p < .05, **p < .01, ***p < .001.

To explore the role of OTUB2 in intestinal inflammation, we induced experimental colitis in *Otub2* ^+/+^ and *Otub2* ^–/–^ mice with DSS. Treatment with DSS induced body weight loss, stool consistency reduction, and rectal bleeding in both *Otub2* ^+/+^ and *Otub2* ^–/–^ mice (Figure 2A and B). However, body weight loss and disease activity were more severe in *Otub2* ^–/–^ mice in comparison to *Otub2* ^+/+^ mice (Figure 2A and B). Correspondingly, after DSS treatment, the colons of *Otub2* ^–/–^ mice were significantly shorter than those of *Otub2* ^+/+^ mice (Figure 2C and D). Moreover, TUNEL staining showed that OTUB2 deficiency increased cell apoptosis in the intestinal mucosa of DSS‐treated mice (Figure 2E). In addition, histopathological examination revealed that OTUB2 deletion exacerbated DSS‐induced mucosal injury and leukocyte infiltration in the colon (Figure 2F and G). PAS/AB staining also revealed that *Otub2* ^–/–^ mice lost more goblet cells than did *Otub2* ^+/+^ mice during experimental colitis (Figure 2G). Consistently, higher levels of pro‐inflammatory *Il1b*, *Il6*, *Tnf*, *Cxcl2* and *Cxcl10* mRNA were detected in the colon of *Otub2* ^–/–^ mice, further consolidating the more severe colitis in these mice (Figure 2H). Given that macrophages are a predominant source of these pro‐inflammatory cytokines in the intestinal mucosa, we analysed cytokine production in lamina propria macrophages by flow cytometry. Indeed, OTUB2 deficiency significantly enhanced the production of TNF‐α, a detrimental cytokine underlying mucosal damage in IBD, in mucosa‐infiltrating macrophages after DSS treatment (Figure S3). In addition to DSS‐induced colitis, OTUB2 ablation also exacerbated experimental colitis induced by TNBS (Figure S4). Collectively, these data show that deletion of OTUB2 exacerbates experimental colitis in mice, indicative of a protective role of OTUB2 in intestinal inflammation.

### 3.3 Haematopoietic cell‐derived OTUB2 alleviates DSS‐induced colitis

**FIGURE 3:**
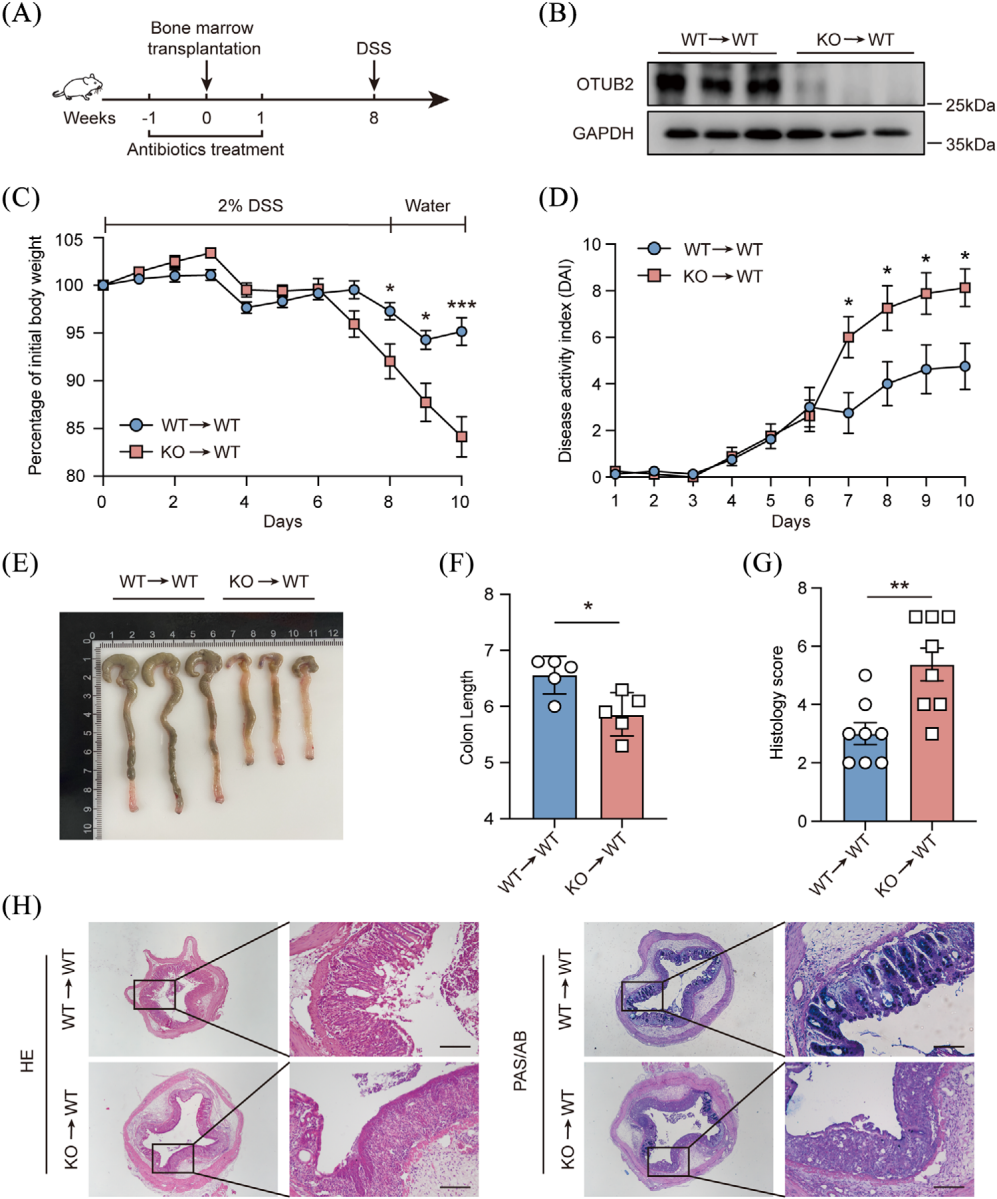
Deficiency of OTUB2 in haematopoietic cells exacerbates DSS‐induced colitis. (A) Experimental flowchart for bone marrow transplantation. (B) Eight weeks after bone marrow transplantation, OTUB2 expression in splenocytes of *Otub2* ^+/+^ mice receiving bone marrow from *Otub2* ^+/+^ (WT→WT) and *Otub2* ^–/–^ (KO→WT) mice was analysed by Western blot. (C, D) The chimeric mice were fed drinking water containing 2% DSS for 8 days, and then given normal drinking water for 2 days. Body weight (C) and disease activity index (D) were recorded daily (*n* = 7/group). (E, F) The representative image (E) and length (F) of colons from chimeric mice on day 10 after DSS treatment. (G and H) Histology score (G) as well as representative H&E and PAS/AB staining (H) of colons from chimeric mice on day 10 after DSS treatment. Scale bar = 50 µm. Data in C, D, F and G are shown as mean ± SEM. *p < .05, **p < .01, ***p < .001.

Given that intestinal epithelial cells (IECs) form the critical intestinal barrier that segregates intestinal microbes and IECs undergo programmed cell death during colitis,^8^, ^28^ OTUB2 may affect experimental colitis by regulating IECs. However, the deletion of OTUB2 did not reduce the production of tight junction proteins in IECs (Figure S5A and B). Besides, OTUB2 deficiency had a negligible effect on the apoptosis of IECs upon stimulation with CHX plus TNF‐α (Figure S5C). To confirm whether OTUB2 exerts its function in colitis by regulating IECs or immune cells, we constructed bone marrow chimeric mice by reconstituting irradiated *Otub2* ^+/+^ mice with bone marrow from *Otub2* ^+/+^ or *Otub2* ^–/–^ mice (Figure 3A and B). Irradiated *Otub2* ^+/+^ mice receiving *Otub2* ^–/–^ bone marrow (KO → WT) were more sensitive to DSS‐induced experimental colitis than irradiated *Otub2* ^+/+^ mice reconstituted with *Otub2* ^+/+^ bone marrow (WT → WT), as shown by the increased body weight loss and disease activity in KO → WT mice (Figure 3C and D). In addition, in comparison to WT → WT mice, KO → WT mice had significantly shorter colons and bigger spleens after DSS treatment (Figures 3E and F and S6). In line with the reduced colon length, H&E and PAS/AB staining found that mice with OTUB2 deficiency in haematopoietic cells had increased epithelium damage, leukocyte infiltration and goblet cell loss in the colonic mucosa (Figure 3G and H). Together, these findings show that the expression of OTUB2 in haematopoietic cells plays a critical role in suppressing colonic inflammation, implying that OTUB2 ameliorates colitis by regulating leukocytes.

### 3.4 OTUB2 is highly expressed in macrophages and regulates macrophage response to MDP

**FIGURE 4:**
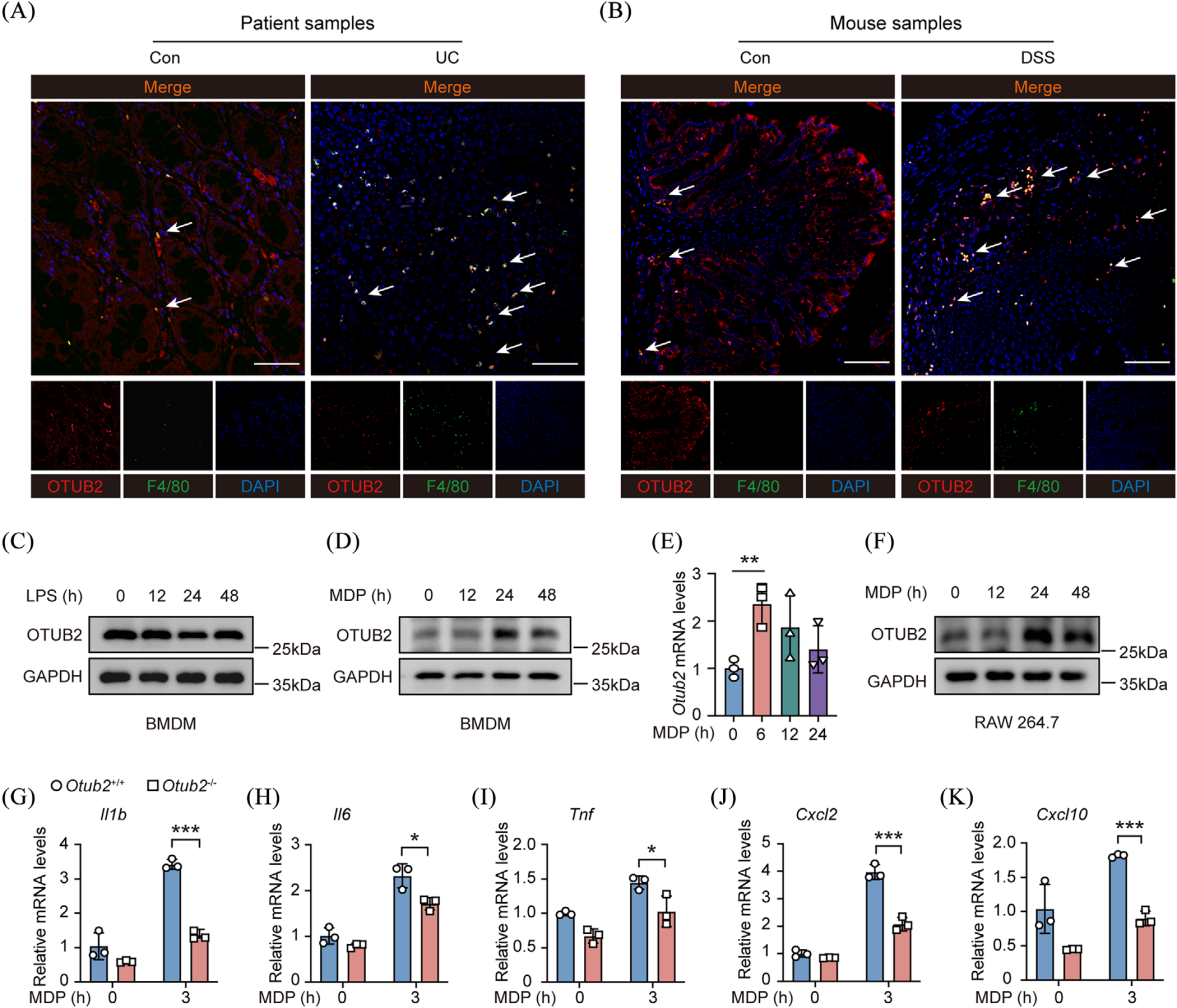
OTUB2 enhances MDP‐induced cytokine production in macrophages. (A, B) Representative OTUB2 (red) and F4/80 (green) immunofluorescence staining of colon samples from humans (A) and mice (B). Scale bar = 100 µm. (C, D) BMDM isolated from C57BL/6 mice were stimulated with 500 ng/mL LPS (C) or 200 ng/mL L18‐MDP (D) for indicated periods of time. OTUB2 protein levels were then analysed by Western blot. (E) BMDM isolated from C57BL/6 mice were stimulated with 200 ng/mL L18‐MDP for indicated periods of time. The relative mRNA levels of *Otub2* were determined by qRT‐PCR. Data are presented as the relative increase over untreated control samples. (F) RAW264.7 cells were stimulated with 200 ng/mL L18‐MDP for indicated periods of time. OTUB2 protein levels were then analysed by Western blot. (G–K) BMDMs isolated from *Otub2* ^+/+^ and *Otub2* ^–/–^ mice were stimulated with 200 ng/mL L18‐MDP for 3 h or left untreated. The relative expression of *Ilb* (G), *Il6* (H), *Tnf* (I), *Cxcl2* (J) and *Cxcl10* (K) mRNA was determined by qRT‐PCR. Data are presented as the relative increase over untreated control samples. Data in E and G–K are displayed as mean ± SEM. *p < .05, **p < .01, ***p < .001.

Among leukocytes, macrophages play an essential role in intestinal homeostasis and inflammation.^6^, ^8^, ^29^ In homeostatic colons of humans and mice, OTUB2 was expressed in both IECs and macrophages, with higher levels of OTUB2 detected in macrophages (Figure 4A and B). In good agreement with previous findings (Figure 1A and B), OTUB2 was markedly downregulated in IECs in inflamed colons. In contrast, the colon‐infiltrating macrophages became the predominant cell population that expressed OTUB2 in inflamed colons of both humans and mice (Figure 4A and B). Macrophages sense and respond to luminal microbes mainly through pattern recognition receptors including TLR4 and NOD2. Lipopolysaccharide (LPS), a specific TLR4 ligand, did not alter OTUB2 expression in macrophages (Figure 4C), and OTUB2 deletion had no impact on LPS‐induced cytokine production (Figure S7). In contrast, MDP, a peptidoglycan component that specifically activates NOD2, induced the upregulation of OTUB2 in primary macrophages and RAW264.7 cells (Figure 4D–F). OTUB2 and OTUB1 are two otubains sharing similar domain architecture (Figure S8A); however, MDP did not induce OTUB1 expression in macrophages (Figure S8B), indicating that the two otubains play distinctive roles in response to MDP. Upon MDP stimulation, OTUB2 deletion significantly reduced the transcription of cytokines and chemokines in macrophages (Figure 4G–K). Noteworthy, NOD2‐induced physiological inflammation is critical for the detection and clearance of bacteria, and loss‐of‐function mutations of *NOD2* are the highest genetic risk for IBD.^8^, ^30^, ^31^, ^32^ In aggregate, these results demonstrate that OTUB2 promotes MDP‐induced innate immune responses in macrophages, indicating that OTUB2 ameliorates colitis by bolstering NOD2‐mediated protective effects in macrophages.

### 3.5 OTUB2 sustains MDP‐induced signalling by stabilising RIPK2

**FIGURE 5:**
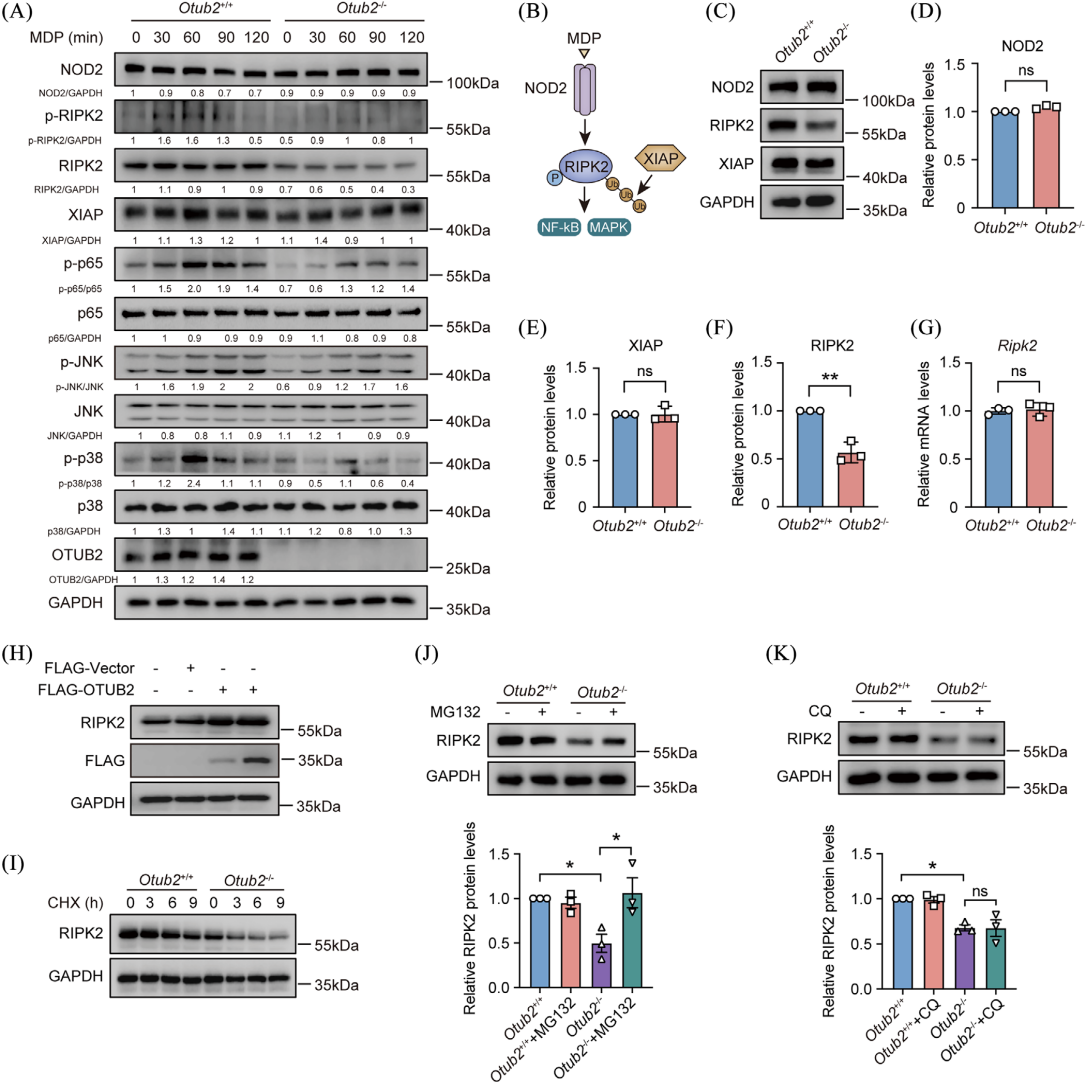
OTUB2 enhances MDP‐induced signalling by inhibiting the proteasomal degradation of RIPK2. (A) After stimulation with 200 ng/mL L18‐MDP for indicated periods of time, BMDMs derived from *Otub2* ^+/+^ and *Otub2* ^–/–^ mice were lysed and analysed by Western blot with indicated antibodies. Densitometric quantification is shown in the figure. (B) Schematic diagram of MDP‐induced signalling. (C) The protein abundance of NOD2, RIPK2 and XIAP in *Otub2* ^+/+^ and *Otub2* ^–/–^ BMDMs was determined by Western blot. (D–F) The relative protein levels of NOD2 (D), XIAP (E) and RIPK2 (F) in *Otub2* ^+/+^ and *Otub2* ^–/–^ BMDMs. (G) The relative mRNA levels of *Ripk2* in *Otub2* ^+/+^ and *Otub2* ^–/–^ BMDMs were determined by qRT‐PCR. (H) RAW264.7 cells were transfected with FLAG‐Vector or FLAG‐OTUB2 plasmids for 24 h. Thereafter, cells were lysed and analysed by Western blot with indicated antibodies. (I) After treatment with 20 ng/mL CHX for indicated periods of time, BMDMs isolated from *Otub2* ^+/+^ and *Otub2* ^–/–^ mice were lysed and analysed by Western blot with indicated antibodies. (J, K) BMDMs isolated from *Otub2* ^+/+^ and *Otub2* ^–/–^ mice were treated with 20 µM MG132 (J) or 50 µM CQ (K) for 6 h or left untreated. Whole Cell lysates were analysed by Western blot with indicated antibodies. Representative immunoblots (upper panel) and quantification (lower panel) are shown. Data in D–G, J and K are shown as mean ± SEM. ns, no significant difference. *p < .05, **p < .01.

To explore the molecular mechanism by which OTUB2 promotes MDP‐induced protective effects in macrophages, we stimulated BMDMs from *Otub2* ^+/+^ and *Otub2* ^–/–^ mice with MDP and then analysed signalling molecules. Phosphorylation of p65 NF‐κB and MAPKs was markedly diminished in the absence of OTUB2 (Figure 5A), explaining the reduced transcription of cytokines and chemokines in *Otub2* ^–/–^ BMDMs after MDP stimulation (Figure 4G–K). Upstream of NF‐κB and MAPKs, MDP activates the proximal signalling complex comprising NOD2, RIPK2, and XIAP (Figure 5B). Interestingly, we found that both the phosphorylation and protein abundance of RIPK2 was reduced in *Otub2* ^–/–^ BMDMs (Figure 5A), indicating that OTUB2 promotes NOD2 signalling by upregulating RIPK2 expression. In resting cells, the deletion of OTUB2 significantly reduced RIPK2 protein levels but had no impact on the protein abundance of NOD2 and XIAP (Figure 5C–F). However, *Otub2* ^+/+^ and *Otub2* ^–/–^ BMDMs had comparable levels of *Ripk2* mRNA (Figure 5G), showing that OTUB2 increases RIPK2 protein abundance in a transcription‐independent way. Consistently, overexpression of OTUB2 upregulated RIPK2 protein levels (Figure 5H). Considering that (i) OTUB2 is a DUB that can stabilise protein substrates,^23^, ^25^ (ii) OTUB2 increases RIPK2 protein levels, (iii) OTUB2 does not affect the de novo synthesis of OTUB2, it is possible that OTUB2 increases RIPK2 protein abundance by inhibiting the degradation of RIPK2 protein. Indeed, in the CHX chase assay, the degradation of RIPK2 was accelerated in the absence of OTUB2 (Figures 5I and S9). The proteasome inhibitor MG132, rather than the lysosome inhibitor CQ, upregulated RIPK2 protein abundance in *Otub2* ^–/–^ BMDMs (Figure 5J and K), indicating that OTUB2 mainly inhibits the proteasomal degradation of RIPK2. Collectively, these data show that OTUB2 promotes MDP‐induced, NOD2‐mediated signalling by inhibiting the proteasomal degradation of RIPK2.

### 3.6 OTUB2 reduces K48 ubiquitination on RIPK2 through its DUB activity

**FIGURE 6:**
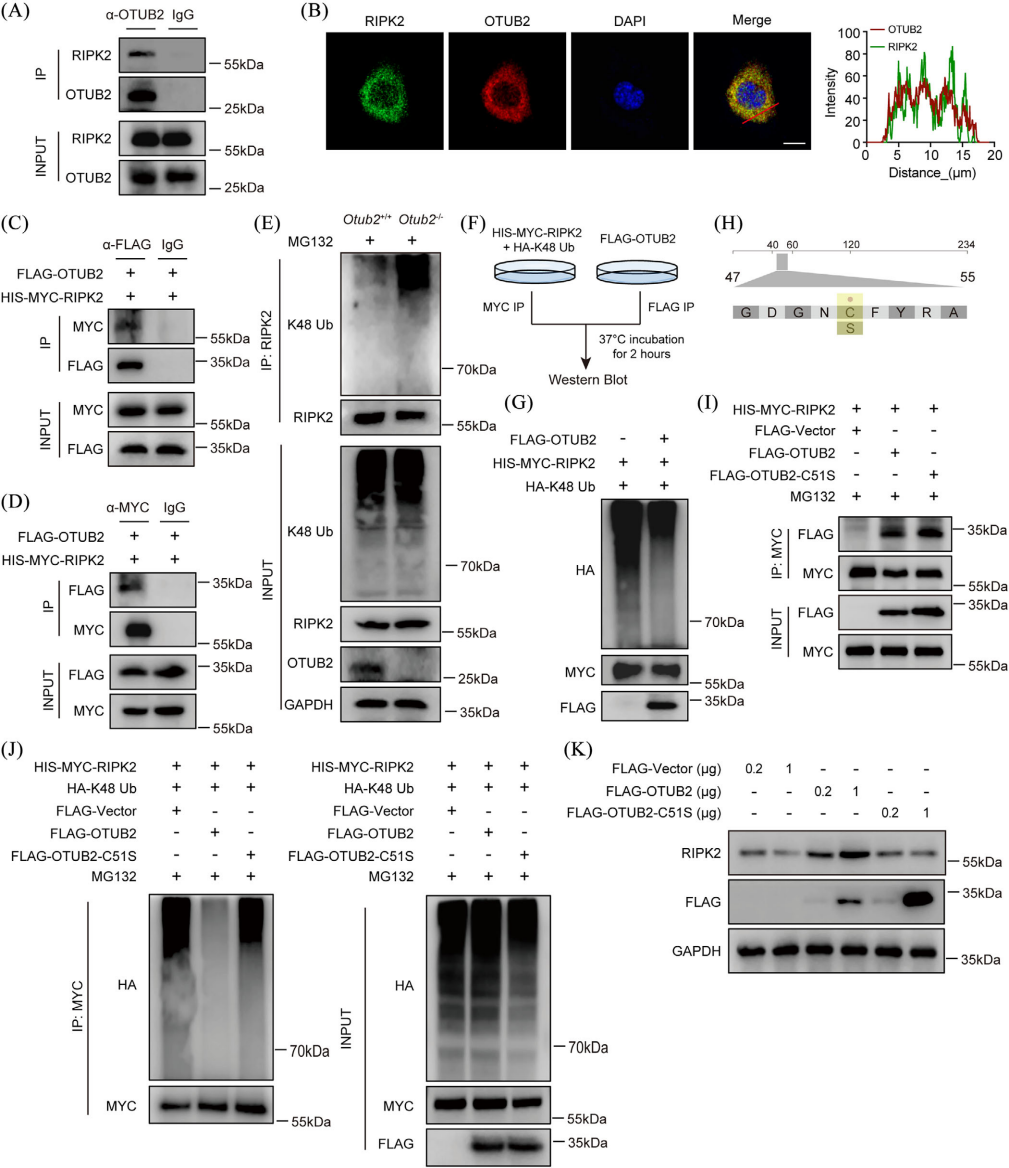
OTUB2 interacts with RIPK2 to mediate K48 deubiquitination. (A) BMDM lysates were immunoprecipitated with anti‐OTUB2 antibody. Immunoprecipitated proteins and cell lysates were analysed by Western blot with indicated antibodies. (B) Subcellular distribution of OTUB2 (red) and RIPK2 (green) in BMDMs was examined by immunofluorescence. Scale bar = 5 µm. (C, D) NIH/3T3 cells were co‐transfected with FLAG‐OTUB2 and HIS‐MYC‐RIPK2 plasmids for 24 h. Whole‐cell lysates were immunoprecipitated with anti‐FLAG (C) or anti‐MYC (D) antibodies. Immunoprecipitated proteins and cell lysates were analysed by Western blot with the indicated antibodies. (E) BMDMs were treated with 20 µM MG132 for 6 h before lysis. Proteins were immunoprecipitated from whole‐cell lysates with anti‐RIPK2 antibody and analysed by Western blot. (F, G) Schematic diagram (F) and representative immunoblots (G) of the in vitro deubiquitination assay. (H) Schematic diagram of the OTUB2 active site and the C51S mutant construct. (I, J) NIH/3T3 cells were transfected with the indicated plasmids for 24 h, followed by treatment with 20 µM MG132 for 6 h. Proteins were immunoprecipitated from whole‐cell lysates with anti‐MYC antibody and analysed by Western blot. (K) NIH/3T3 cells were transfected with the indicated plasmids for 24 h. Whole‐cell lysates were analysed by Western blot with the indicated antibodies.

To determine the mechanism how OTUB2 stabilises RIPK2, we then examined whether OTUB2 directly interacted with RIPK2. RIPK2 was detected in the immunocomplex harvested from BMDMs with anti‐OTUB2 antibody (Figure 6A), showing that RIPK2 interacts with OTUB2. In addition, immunofluorescence staining revealed that OTUB2 and RIPK2 co‐localised in the cytoplasm of BMDMs (Figure 6B). Moreover, we found that exogenously expressed FLAG‐OTUB2 could bind with HIS‐MYC‐RIPK2 (Figure 6C and D), confirming the direct interaction between the two proteins. Deletion of OTUB2 increased the K48 ubiquitination of RIPK2 (Figure 6E), indicating that OTUB2 stabilises RIPK2 by reducing the K48 polyubiquitination of RIPK2. Indeed, OTUB2 could efficiently remove already established K48 polyubiquitination chains on RIPK2 in the in vitro deubiquitination assay (Figure 6F and G). The DUB activity of OTUB2 is exerted by the C51 residue (Figure 6H). Of note, the other otubain, OTUB1, reduces the ubiquitination of multiple substrates in a DUB activity‐independent way.^33^, ^34^ To test whether the DUB activity of OTUB2 is required for K48 deubiquitinating RIPK2, we constructed the OTUB2 C51S mutant, which lacked the DUB activity (Figure 6H). Although the C51S mutant could interact with RIPK2 (Figure 6I), it failed to deubiquitinate and stabilise RIPK2 (Figure 6J and K). Given that RIPK2 is essential for NOD2 signalling and that OTUB2 stabilised RIPK2 through the C51 active site, overexpression of OTUB2, rather than the C51S mutant, increased the transcription of *Il1b*, *Il6* and *Tnf* in RAW264.7 cells upon MDP stimulation (Figure S10). Moreover, RIPK2 overexpression increased MDP‐induced cytokine production in both OTUB2‐ and OTUB2 C51S‐overexpressed cells, and abolished the differences between the two groups (Figure S10). Taken together, these results demonstrate that OTUB2 regulates the K48 ubiquitination and stability of RIPK2 through the active site C51.

### 3.7 OTUB2 deletion abolishes the protective effects of MDP in colitis

**FIGURE 7:**
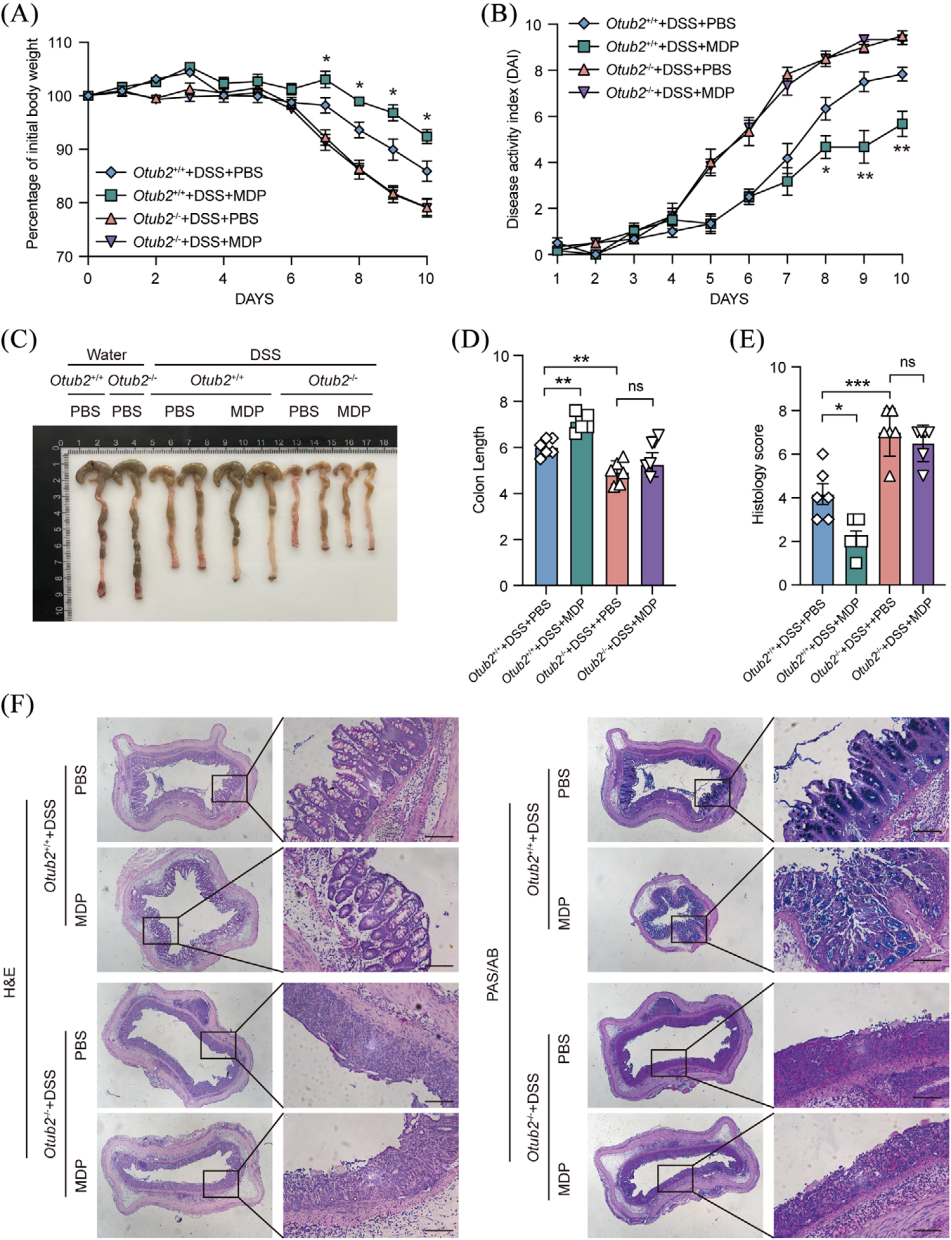
MDP treatment alleviates DSS‐induced colitis in *Otub2* ^+/+^ mice but not in *Otub2* ^–/–^ mice. (A) *Otub2* ^+/+^ and *Otub2* ^–/–^ mice were fed drinking water containing 2% DSS for 7 days and subsequently given regular drinking water for 3 days. In addition, mice were intraperitoneally injected with either MDP (100 µg; 200 µL/mouse) or 200 µL of PBS on days 0, 1 and 2. Body weight (A) and disease activity index (B) were recorded daily (*n* = 6/group). (C, D) The representative image (C) and length (D) of colons from *Otub2* ^+/+^ and *Otub2* ^–/–^ mice on day 10 after DSS treatment. (E, F) Histology score (E) as well as representative H&E and PAS/AB staining (F) of colons from *Otub2* ^+/+^ and *Otub2* ^–/–^ mice on day 10 after DSS treatment. Scale bar = 50 µm. Data in A, B, D and E are shown as mean ± SEM. ns, no significant difference. *p < .05, **p < .01, ***p < .001.

Considering that (i) the NOD2‐RIPK2 signalling is protective in IBD,^30^, ^31^, ^35^ (ii) MDP treatment activates the NOD2‐RIPK2 signalling and protects mice from experimental colitis,^32^, ^36^, ^37^ and (iii) we previously found OTUB2 enhanced NOD2 signalling by stabilising RIPK2, we next addressed the significance of OTUB2 in NOD2 signalling in vivo by investigating the effect of MDP treatment on experimental colitis in *Otub2* ^+/+^ and *Otub2* ^–/–^ mice. MDP administration significantly ameliorated colitis in *Otub2* ^+/+^ mice with a concomitant reduction in body weight loss, disease activity and colon shortening (Figure 7A–D). In addition, MDP administration also attenuated epithelium damage, leukocyte infiltration and goblet cell loss in the colon of *Otub2* ^+/+^ mice (Figure 7E and F). However, these protective effects of MDP were absent in *Otub2* ^–/–^ mice (Figure 7A–F), indicating that OTUB2 is essential for NOD2‐mediated protective effects in colitis. Altogether, these results show that OTUB2 sustains the ability of the NOD2‐RIPK2 signalling complex to preserve intestinal homeostasis and control colonic inflammation.

## 4 DISCUSSION

NOD2 plays a key role in intestinal homeostasis and loss‐of‐function mutations of *NOD2* are closely associated with IBD.^30^, ^31^ In this study, we found that NOD2 activation induced the upregulation of the DUB OTUB2. Furthermore, OTUB2 enhanced NOD2‐mediated signalling and protective effects in intestinal inflammation by stabilising RIPK2. Thus, OTUB2 serves as a key enzyme safeguarding intestinal homeostasis.

NOD2 is an intracellular pattern recognition receptor (PRR) that senses intracellular pathogen‐associated molecular patterns (PAMPs). NOD2 specifically recognises MDP, a component of bacterial peptidoglycan conserved in both Gram‐positive and ‐negative bacteria.^38^, ^39^ Upon activation by MDP, NOD2 undergoes oligomerisation and then recruits RIPK2 by homophilic domain interactions. Subsequently, activated RIPK2 promotes the recruitment and activation of TAK1, which further activates MAPK and NF‐κB signalling pathways to produce cytokines and antimicrobial peptides.^40^ The physiological inflammatory program initiated by NOD2 creates a dynamic balance between immune factors and intestinal microbiota, thereby shielding the intestinal homeostasis. Ubiquitination critically regulates apical NOD2 signalling by controlling the activation and degradation of NOD2 and RIPK2.^41^, ^42^, ^43^ The activity and antibacterial function of NOD2 requires its K63 ubiquitination.^41^ Similarly, K63 and M1 polyubiquitination is essential for proper RIPK2 function in NOD2 signalling.^32^, ^41^, ^42^, ^43^, ^44^, ^45^ In contrast, K48‐specific ubiquitination induces the proteasomal degradation of NOD2 and RIPK2, serving as a mechanism regulating hyporesponsiveness and tolerance to MDP.^46^, ^47^ In this study, we found that deletion of OTUB2 diminished MDP‐induced signal transduction and cytokine production by specifically reducing the protein abundance of RIPK2, but not NOD2. OTUB2 removes K48‐linked polyubiquitin chains from RIPK2 to inhibit its degradation via the 26S proteasome, thereby stabilising RIPK2 protein for efficient signal transduction. Moreover, the catalytically inactive C51S mutant of OTUB2 failed to deubiquitinate and stabilise RIPK2, indicating that OTUB2 regulates NOD2 signalling in a DUB activity‐dependent way.

Mucosal immunity in the intestine is orchestrated by both immune cells and IECs, and the DUB A20 affects colitis by regulating cell death of IECs.^14^, ^15^ However, OTUB2 deficiency did not influence IEC apoptosis, ruling out the possibility that OTUB2 affects intestinal inflammation by regulating IECs. Given that (i) OTUB2 was highly expressed in macrophages in the colon, (ii) OTUB2 in haematopoietic cells played a predominant role in colitis, and (iii) OTUB2 enhanced MDP‐induced cytokine production in macrophages, we concluded that OTUB2 exerted its function in intestinal inflammation by regulating macrophages.

The two otubains, that is, OTUB1 and OTUB2, have considerable structural overlap and sequence conservation.^26^, ^48^ Despite this, OTUB1 and OTUB2 are distinctive in substrate specificity and ubiquitin cleavage.^49^ Only the expression of OTUB2, but not OTUB1, was induced by MDP treatment, indicative of a specific involvement of OTUB2 in NOD2 signalling. Unlike OTUB1, which has been extensively studied in a plethora of diseases, the in vivo function of OTUB2 remains largely unknown.^50^, ^51^ In this report, we demonstrated that OTUB2 ameliorated colitis, providing novel insights into the pathophysiological function of OTUB2.

Polymorphisms and mutations in *NOD2* represent the most replicated genetic associations with IBD, however, most of IBD patients bear wild‐type NOD2, implying that signalling molecules downstream of NOD2 may be defective in those patients. Although existing studies did not show a clear association of any polymorphisms or mutations in *Otub2* with IBD, it cannot rule out the possibility that certain variants of *Otub2* may contribute to IBD susceptibility. In summary, our data demonstrate that OTUB2 ameliorates intestinal inflammation by preserving the protective NOD2 signalling in macrophages through deubiquitinating and stabilising RIPK2. This study identifies OTUB2 as a key factor regulating intestinal homeostasis and inflammation, providing new therapeutic opportunities for the treatment of intestinal inflammation.

## AUTHOR CONTRIBUTIONS

Xu Wang conceptualisation; Xue Du, Jun Xu, Fuqi Mei, Jiangyun Shen, Bincheng Zhou, Zhenhu Zhu, Dirk Schlüter, Jing Ruan and Xu Wang data collection and analysis; Zhongding Li, Xian Su. and Jianmin Li technical support and resources; Xue Du and Xu Wang writing – original draft; Xue Du, Dirk Schlüter, Jing Ruan and Xu Wang writing – review and editing; Xu Wang funding acquisition.

## CONFLICT OF INTEREST STATEMENT

The authors declare that they do not have conflicts of interest.

## ETHIC STATEMENT

The study on clinical samples was approved by the Ethics Committee in Clinical Research of the First Affiliated Hospital of Wenzhou Medical University (Approval number: KY2023‐R182). Animal experiments were approved by the Animal Management and Ethics Committee of Wenzhou Medical University (approval number: wydw2023‐0598).

## Supporting information



Supporting information

## Data Availability

Data are included in the article and supplementary materials.
