# Supplementary material for: Deubiquitination of RIPK2 by OTUB2 augments NOD2 signalling and protective effects in intestinal inflammation
Source: Clin Transl Med. 2024 Oct 2;14(10):e70038. doi: 10.1002/ctm2.70038 (PMC11446981; doi:10.1002/ctm2.70038)
Supplement: Supplementary file 1 — Supporting information [file CTM2-14-e70038-s001.docx]

**Deubiquitination of RIPK2 by OTUB2 augments NOD2 signaling and protective effects in intestinal inflammation**

**Supplementary Figures and tables**

**Supplementary Figure 1**


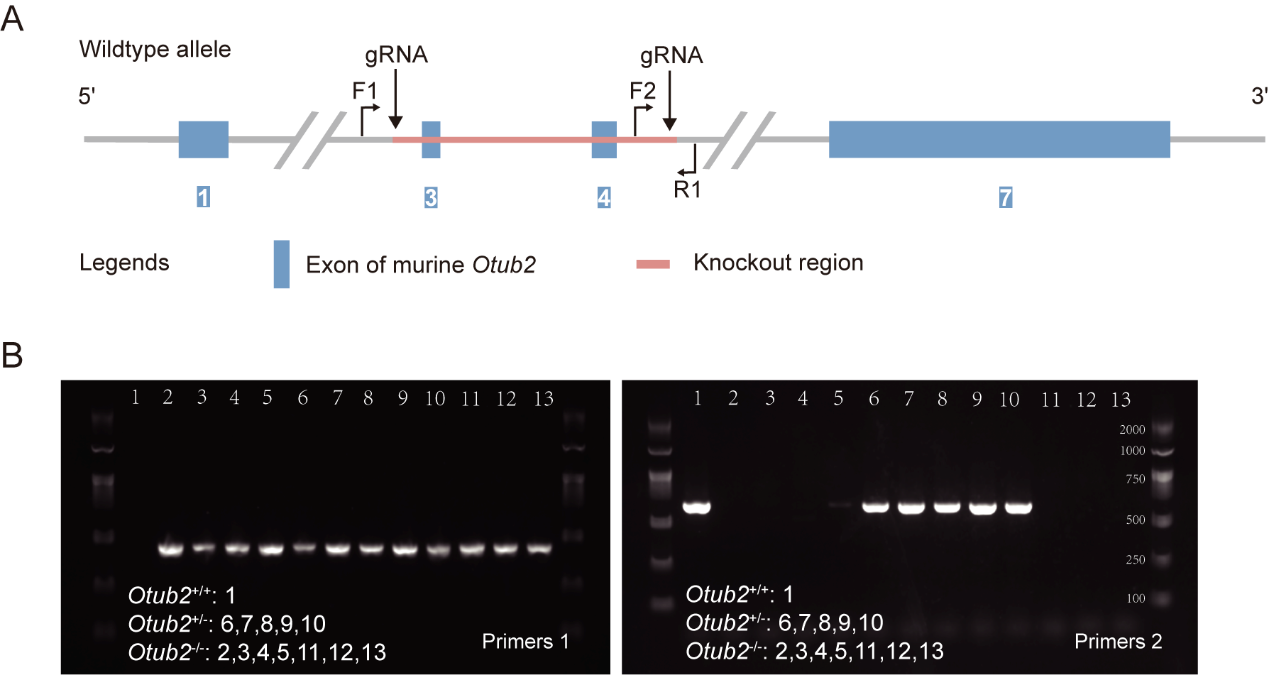


**Supplementary Figure 1. Construction and genotyping of *Otub2*^-/-^ mice.** (A) Construction strategy for the *Otub2* knockout allele. (B) Genotyping of *Otub2*^+/+^, *Otub2*^+/-^, and *Otub2*^-/-^ mice was carried out by PCR with primers shown in (A).

**Supplementary Figure 2**

**
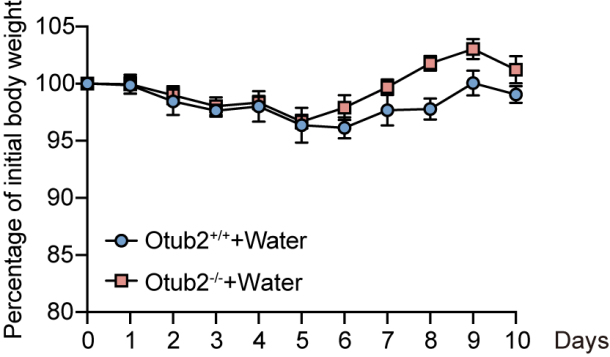
**

**Supplementary Figure 2. *Otub2*^+/+^ and *Otub2*^-/-^ mice receiving regular drinking water have comparable body weight.** *Otub2*^+/+^ and *Otub2*^-/-^ mice were fed regular drinking water for 10 days. Body weight was recorded daily (n = 5/group).

**Supplementary Figure 3**


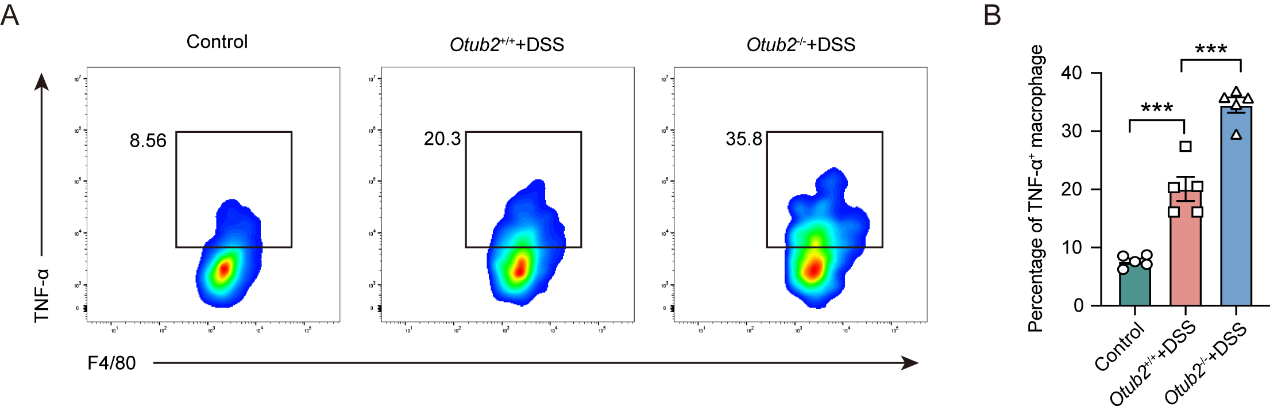


**Supplementary Figure 3. OTUB2 deficiency significantly increases the production of** **TNF-α by macrophages in inflamed mucosa.** (A-B) Representative dot plots (A) and quantification (B) of TNF-α^+^ macrophages infiltrating the intestinal mucosa. Mice in the control group received regular drinking water. Mice in the DSS group were fed 2% DSS for 8 days and then given normal drinking water for 2 days. Mean ± SEM, *** p < 0.001.

**Supplementary Figure 4**

**
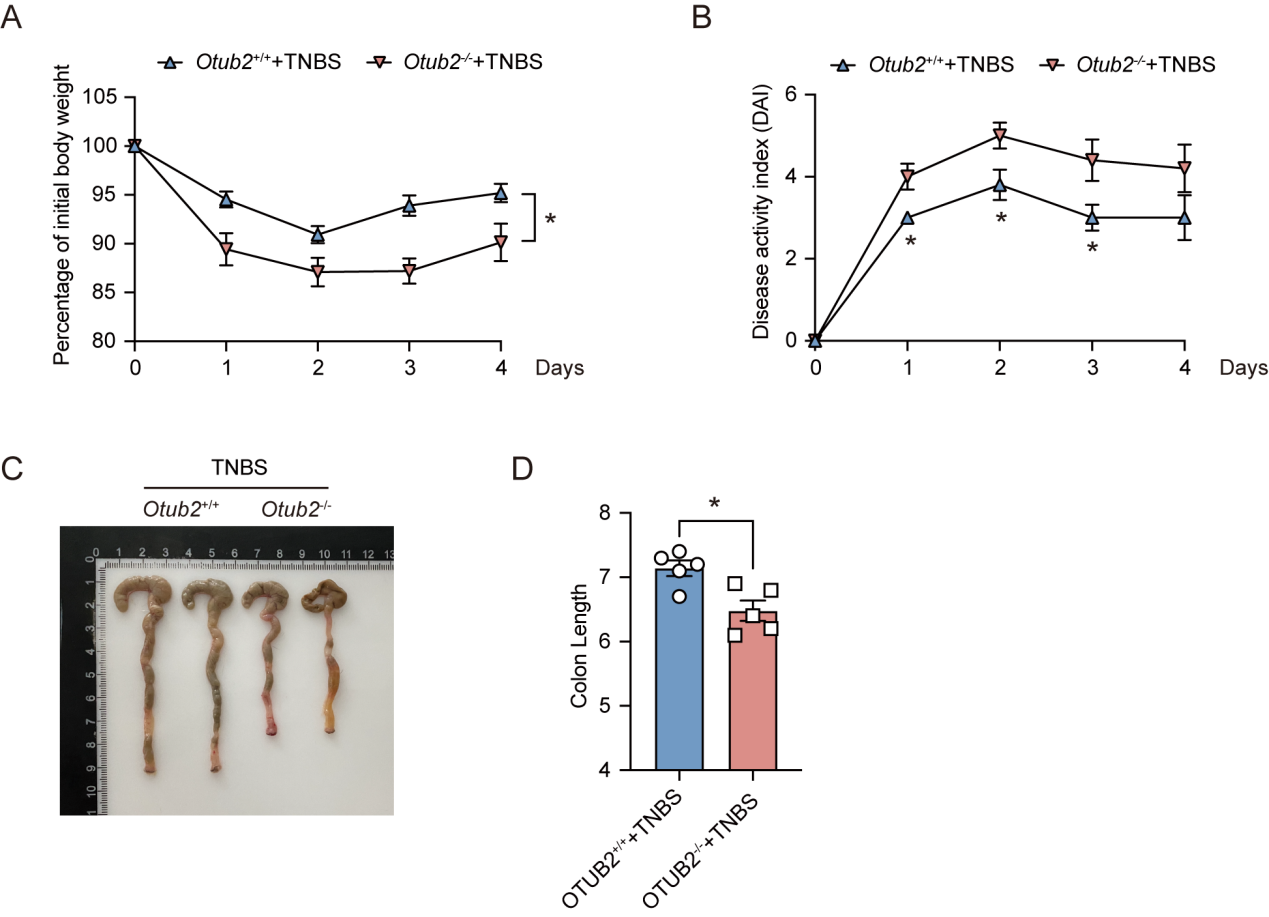
**

**Supplementary Figure 4. OTUB2 deletion exacerbates TNBS-induced colitis.** (A-B) Colitis was induced in *Otub2*^+/+^ and *Otub2*^-/-^ mice with TNBS. Body weight (A) and disease activity index (B) were recorded daily (n = 5/group). (C-D) The representative image (C) and length (D) of colons from *Otub2*^+/+^ and *Otub2*^-/-^ mice on day 4 after TNBS treatment. Data in (A, B, D) are shown as mean ± SEM. * p < 0.05.

**Supplementary Figure 5**


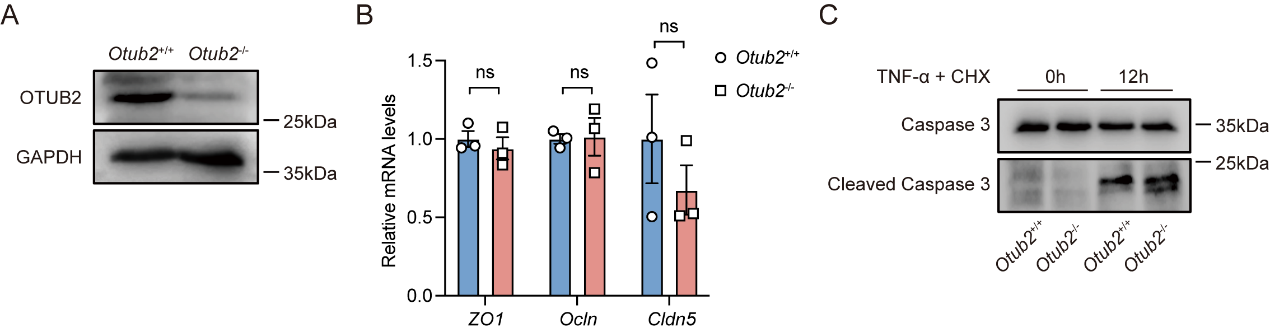


**Supplementary Figure 5. OTUB2 deletion does not affect tight junction protein production and apoptosis of MODE-K cells.** (A) OTUB2 expression in *Otub2*^+/+^ and *Otub2*^-/-^ MODE-K cells was analyzed by Western blot. (B) mRNA levels of *ZO1*, *Ocln*, and *Cldn5* in *Otub2*^+/+^ and *Otub2*^-/-^ MODE-K cells were determined by qRT-PCR. Mean ± SEM, ns, no significant difference. (C) *Otub2*^+/+^ and *Otub2*^-/-^ MODE-K cells were stimulated with TNF-α (40 ng/ml) + CHX (20 μg/ml) for 12 h. Whole-cell lysates were analyzed by Western blot with anti-Caspase 3 antibody.

**Supplementary Figure 6**

**
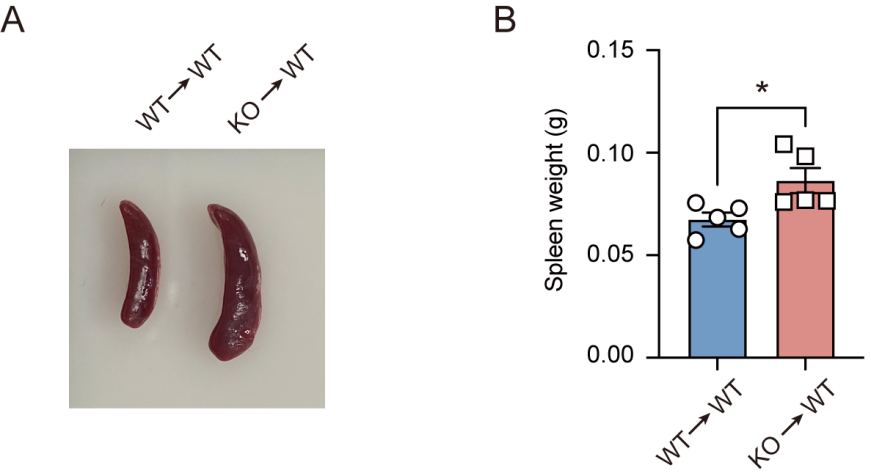
**

**Supplementary Figure 6. Hematopoietic cell-specific deficiency of OTUB2 increases spleen weight after DSS treatment.** (A-B) The representative image (A) and weight (B) of spleens from chimeric mice on day 10 after DSS treatment. Mean ± SEM. * p < 0.05.

**Supplementary Figure 7**

**
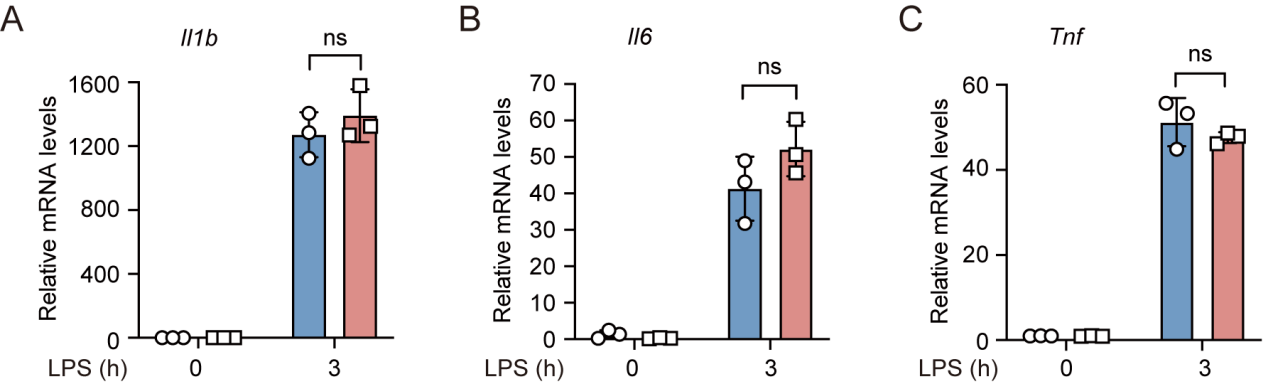
Supplementary Figure 7. OTUB2 deletion has no impact on LPS-induced cytokine production in macrophages.** (A-C) BMDMs isolated from *Otub2*^+/+^ and *Otub2*^-/-^ mice were stimulated with 500 ng/ml LPS for 3 h or left untreated. The mRNA levels of *Il1b* (A), *Il6* (B), and *Tnf* (C) were analyzed by qRT-PCR. ns, no significant difference.

**Supplementary Figure 8**


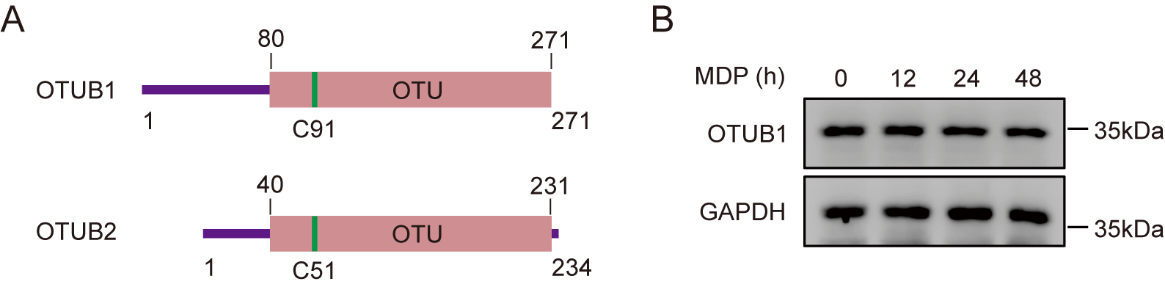


**Supplementary Figure 8. MDP stimulation has no impact on OTUB1 protein levels in BMDMs.** (A) Domain architecture of OTUB1 and OTUB2. (B) BMDMs isolated from C57BL/6 mice were stimulated with L18-MDP (200 ng/ml) for indicated periods of time. OTUB1 protein levels were then analyzed by Western blot.

**Supplementary Figure 9**

**
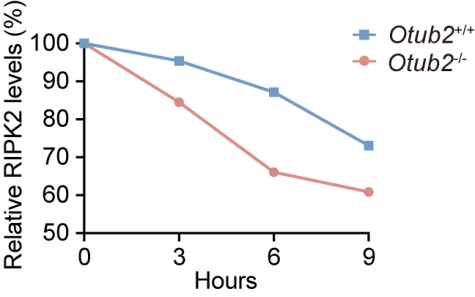
**

**Supplementary Figure 9. OTUB2 deletion accelerates the degradation of RIPK2.** BMDMs isolated from *Otub2*^+/+^ and *Otub2*^-/-^ mice were treated with 20 ng/ml CHX for indicated periods of time, and then analyzed by Western blot to detect RIPK2 protein abundance. The data show the relative protein levels of RIPK2 normalized to GAPDH.

**Supplementary Figure 10**

**
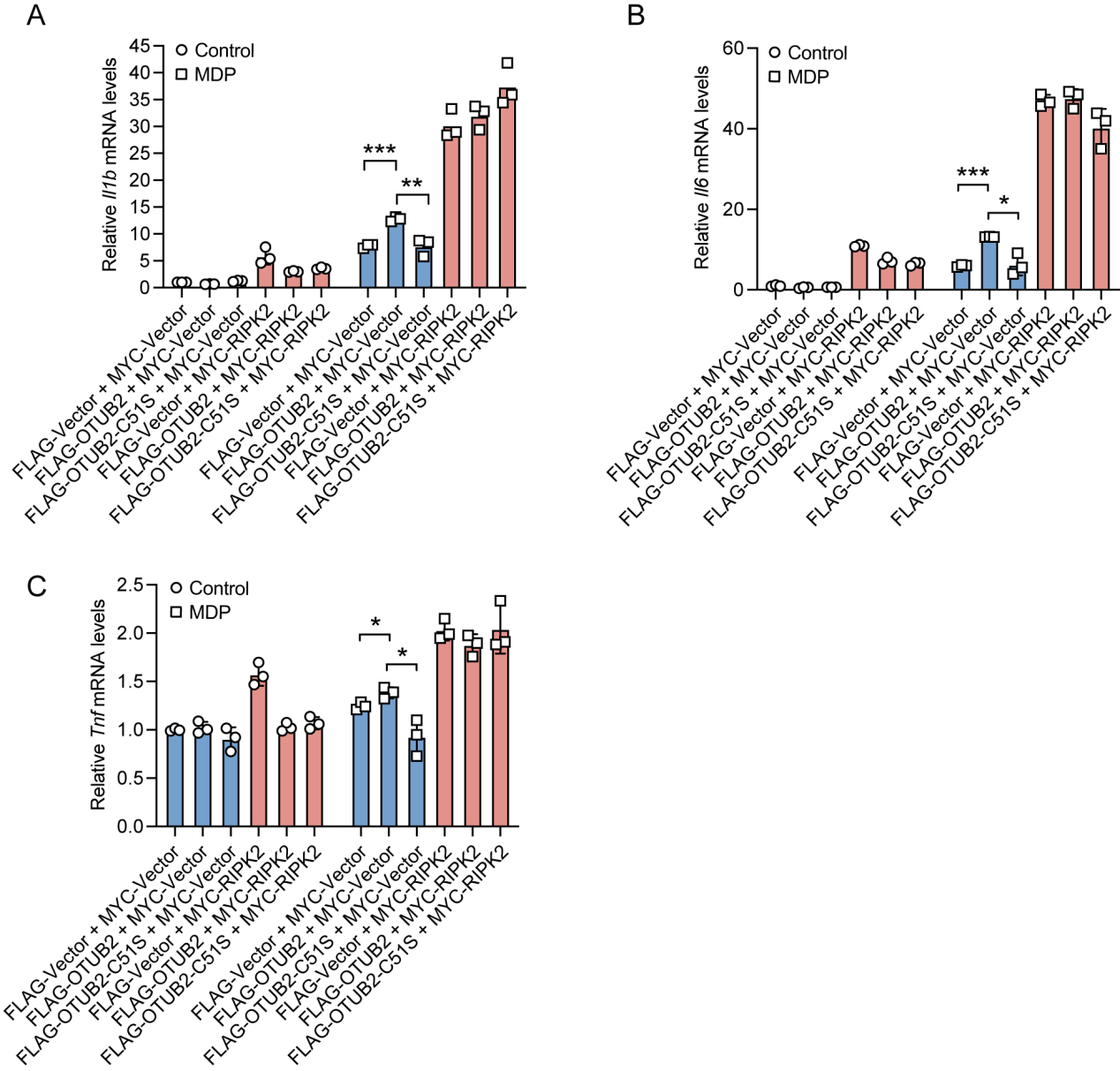
**

**Supplementary Figure 10. The C51 active site is indispensable for OTUB2 to enhance MDP-induced cytokine production.** (A-C) *Otub2*^-/-^ RAW264.7 cells were transfected with indicated plasmids for 24 h. After that, cells were stimulated with L18-MDP (200 ng/ml) for 3 hours or left untreated. The relative mRNA levels of *Il1b* (A), *Il6* (B), and *Tnf* (C) were determined by qRT-PCR. Data are shown as mean ± SEM. * p < 0.05, ** p < 0.01, *** p < 0.001.

**Supplementary Table 1.** qRT-PCR primers

| **Gene** | **Species** | **Forward primer（5'-3'）** | **Reverse primer（5'-3'）** |
| --- | --- | --- | --- |
| *β-actin* | mouse | CCGTGAAAAGATGACCCAGA | TACGACCAGAGGCATACAG |
| *Il-6* | mouse | GAGGATACCACTCCCAACAGACC | AAGTGCATCATCGTTGTTCATACA |
| *Il-1β* | mouse | ACTCCTTAGTCCTCGGCCA | CCATCAGAGGCAAGGAGGAA |
| *Tnf* | mouse | CAGGGGCCACCACGCTCTTC | TTTGTGAGTGTGAGGGTCTGG |
| *Cxcl2* | mouse | GACAGAAGTCATAGCCACTCTCAAG | TCAGTTAGCCTTGCCTTTGTTCAG |
| *Cxcl10* | mouse | CCAAGTGCTGCCGTCATTTTC | GGCTCGCAGGGATGATTTCAA |
| *Otub2* | mouse | GACCTCATTCCTCGCTTCCATCTG | GTGGGTAAGACAAGACGGAGAACAG |
| *Ripk2* | mouse | TCGTGTGGATCCTCTCTGCTCT | TTCCAGGACAGTGGTGTGCCTT |
| *ZO1* | mouse | AGCAGTGGAAGAAGTTACAGTTGAG | AGAAGGGCTGACGGGTAAATCC |
| *Ocln* | mouse | TGAAAGTCCACCTCCTTACAGA | CCGGATAAAAAGAGTACGCTGG |
| *Cldn5* | mouse | GCAAGGTGTATGAATCTGTGCT | GTCAAGGTAACAAAGAGTGCCA |

**Supplementary Table 2.** Basic patient information

| **Colon cancer patients** | Gender | Male | 7 |
| --- | --- | --- | --- |
|  |  | Female | 2 |
|  | Age (Year) | Minimum age | 50 |
|  |  | Maximum age | 85 |
| **UC patients** | Gender | Male | 6 |
|  |  | Female | 1 |
|  | Age (Year) | Minimum age | 19 |
|  |  | Maximum age | 69 |
